# Autonomous Aldosterone Secretion in Patients with Adrenal Incidentaloma

**DOI:** 10.3390/biomedicines10123075

**Published:** 2022-11-30

**Authors:** Piotr Kmieć, Ewa Zalewska, Katarzyna Kunicka, Ewa Świerblewska, Krzysztof Sworczak

**Affiliations:** 1 Department of Endocrinology and Internal Medicine, Medical University of Gdańsk, 17 Smoluchowskiego Street, 80214 Gdańsk, Poland; 2 Department of Hypertension and Diabetology, Medical University of Gdańsk, 17 Smoluchowskiego Street, 80214 Gdańsk, Poland

**Keywords:** aldosterone, renin, hypertension, hyperaldosteronism diagnosis, left ventricular hypertrophy, ambulatory blood pressure monitoring

## Abstract

In recent years, research has emphasized the significance of mild clinical and biochemical presentations of primary aldosteronism (PA) that do not meet current diagnostic criteria of the syndrome. In this study, we assessed the prevalence of autonomous aldosterone (Ald) secretion (AAS), defined as a positive (>1.2 ng/dL/mIU/L) Ald-to-renin ratio (ADRR) combined with unsuppressed Ald (>4 ng/dL), and its associations with blood pressure (BP), cardiac function, and common carotid artery (CCA) intima-media thickness (IMT) in patients with incidentally discovered adrenal adenomas (AI), who were either normo- or hypertensive but had no other cardiovascular disease. Among 332 AI patients hospitalized between November 2018 and December 2019, 63 study participants were recruited (26 normo- and 37 hypertensive), who underwent hormonal examinations, 24 h ambulatory BP measurement, transthoracic echocardiography, and CCA IMT assessment without altering chronic medications. AAS was found in approximately 25% of subjects (seven normo- and nine hypertensive); urinary aldosterone excretion (UAldE) exceeded 10 ug/day in none of the subjects. The left ventricular mass index correlated positively with UAldE in non-diabetic patients (*n* = 50), and negatively with renin in those without beta blocker therapy (*n* = 38). The study shows that a pragmatic approach to hormonal assessment (*n*o chronic therapy modification) may reveal patients with AAS. Screening for this subclinical PA presentation is probably more effective with a permissive ADRR than UAldE in such a setting.

## 1. Introduction

The recommended method of screening for primary aldosteronism (PA) is determining the aldosterone-to-renin ratio [1]. It is acquired by dividing plasma/serum aldosterone (Ald) by either plasma renin activity (PRA) or direct renin concentration (DRC), abbreviated as ARR and ADRR, respectively. However, widely applied ARR/ADRR cutoffs may largely underestimate PA prevalence reflected by 24 h assessment of urinary aldosterone excretion (UAldE) [2]. 

In fact, accumulating evidence indicates the current approach toward PA does not encompass the whole spectrum of renin-independent excess aldosteronism [3,4]. Recently, we proposed the term ‘autonomous aldosterone secretion’ (AAS) to describe biochemical and clinical presentations of the disorder that do not meet current criteria of PA [5]. These presentations include: (1) patients with normotension (*n*T) and normokalemia but biochemically proven Ald excess, and (2) patients with HT and normokalemia who meet PA screening but not confirmatory test criteria, provided Ald is not suppressed (in both cases).

While a fully objective assessment of Ald secretion requires therapy modification to limit the influence on the renin–angiotensin–aldosterone system (RAAS), this requirement frequently dissuades clinicians from ordering screening — research shows less than 10% of eligible patients undergo it both in primary care and specialist settings [6,7]. Furthermore, in overt PA, medication withdrawal is often unnecessary and not advised by some, since, in principle, a constellation of suppressed renin and unsuppressed Ald should not occur in a person with normal RAAS function, regardless of the use of interfering medications [7,8]. What should also be considered in the approach to possible excess aldosteronism is the selection of controls used for the determination of test cutoffs. The inclusion of those with adrenal adenomas or hypertension (HT) most probably underestimates the prevalence of PA [4]. 

In this cross-sectional study, we examined the prevalence of AAS and its associations with blood pressure (BP), cardiac function assessed by transthoracic echocardiography (TTE), and intima-media thickness (IMT) among patients with incidentally discovered adrenal tumors (AI) with radiological features of adrenocortical adenomas and/or hyperplasia, who were either normo- or hypertensive but had no other cardiovascular disease (CVD). 

## 2. Materials and Methods

### 2.1. Participants and Protocol

Subjects were recruited from November 2018 through December 2019 among patients with adrenal incidentalomas (AI), as defined in the European Society of Endocrinology guidelines [9], who were admitted to the Department of Endocrinology and Internal Medicine of the Medical University of Gdańsk hospital for hormonal work-up of the adrenal tumor.

The inclusion criteria were aged above 18, and the presence of an AI with radiological features of an adrenal cortex adenoma or nodular hyperplasia. The exclusion criteria comprised: (1) overt clinical and/or biochemical features of adrenal hormone deficiency or excess other than mild autonomous cortisol secretion (MACS), defined as an 8 a.m. cortisol concentration of between 50 and 140 nmol/L following oral intake of 1 mg of dexamethasone (DXM) at 11 p.m. the preceding evening (i.e. overnight DXM suppression test, 1-mg DST), (2) age over 75 years, (3) therapy with glucocorticoids, mineralocorticoid receptor antagonists (MRAs), intake of non-steroidal anti-inflammatory drugs or licorice in the fortnight preceding hormonal examinations, (4) infection, (5) poorly controlled or other than type 2 diabetes mellitus (T2DM), (7) present and past alcohol abuse, (8) obesity grade 3 (i.e. with a body mass index, BMI, of at least 40 kg/m^2^), (9) CVD such as valvular defects, coronary artery disease, heart failure, atrial fibrillation, history of stroke or transient ischemic attack, peripheral artery disease, (10) active malignancy, (11) decompensated autoimmune disease, as well as an autoimmune disease associated with cardiovascular and/or renal complications, (12) estimated glomerular filtration rate (eGFR) below 60 mL/min/1.73 m^2^, (13) lack of consent for participation or withdrawal thereof, and (14) normokaliemia. Initially, out of 332 consecutive patients with an AI, 73 were recruited, since the others met at least one exclusion criterion. A further 10 patients were excluded: four due to abnormal TTE, i.e., regional left ventricular (LV) wall motion abnormalities suggestive of coronary artery disease, two due to evidence of overt PA (confirmed by a seated saline suppression test), two due to withdrawal of consent, one due to suspicion of adrenal insufficiency, and one with a final diagnosis of a pheochromocytoma. Therefore, 63 patients were analyzed. 

The study protocol consisted of: recording patient history and physical examination, ECG, laboratory examinations, 24 h ambulatory blood pressure monitoring (ABPM), TTE, and measurement of common carotid arteries’ (CCAs) IMT. As previously reviewed, PA/AAS has been associated with HT, atherosclerosis, metabolic syndrome, and pathologic cardiac remodeling, which underlies the methodology of the study (respectively, ABPM, IMT, lipid profile, and TTE) [5,7,10].

Apart from the 1 mg DST, no dynamic tests were performed. AAS was stated in: (a) hypertensive patients with a positive ADRR (>1.2 ng/dL/mIU/L), and Ald > 4 ng/dL (unsuppressed), provided UAldE was <12 ug/d (threshold indicative of overt PA); and (b) patients with NT, a positive ADRR and unsuppressed Ald, and/or UAldE > 10 ug/d.

### 2.2. Laboratory Examinations

All study participants underwent the following hormonal work-up: 8 a.m. serum/plasma cortisol, ACTH, Ald, DRC, dehydroepiandrosterone sulfate (DHEA-S), natrium (*n*a), potassium (K), 8 p.m. cortisol, 8 a.m. post 1 mg DST cortisol, 24 h urinary excretion of: free cortisol (UFC), aldosterone, natrium (UNaE), potassium, as well as meta- and normetanephrines, and 3-metoxy-thyramine. Morning baseline blood samples were drawn after a night’s rest and two hours of upright posture (seated position 5–15 min before venipuncture). The following were also examined: high-sensitivity CRP, TSH, lipid profile, and 24 h albumin excretion. Laboratory examinations were performed in the Central Diagnostic Laboratory of the same hospital using a Siemens IMMULITE 1000 Immunoassay System (most biochemical tests) and Abbott Architect analyzer (spectrophotometric method). Ald and DRC were determined by immunochemiluminescence using DiaSorin assays. Reference ranges recommended by manufacturers were adopted as normal. Due to a lack of local ADRR thresholds, in accordance with a recent consensus statement, a permissive cutoff of 1.2 ng/mL/mIU/L was adopted (ADRR is further given without units) [11]. Ald of at least 4 ng/dL was considered unsuppressed.

### 2.3. Ambulatory Blood Pressure Monitoring

ABPM was conducted for at least 24 h with a Spacelabs Ontrak 90227 monitor on the non-dominant arm. BP was recorded every 20 min during daytime and every 30 min at night. Patients were obligated to follow an arbitrary period between 10 p.m. and 6 a.m. for nighttime rest. If less than 70% of measurements were valid or fewer than seven valid measurements for nighttime were available, ABPM was repeated or not included in the analyses. Results included mean 24 h, diurnal and nocturnal SBP and DBP, as well as dipping status (abnormal if BP did not lower or lowered by less than 10% during nighttime versus daytime, i.e., non-dipper status). Normal mean SBP/DBP values were adopted from the European Society of Cardiology/European Society of Hypertension 2018 guidelines: below 130/80 mmHg for the 24 h period, below 135/85 mmHg for daytime, and below 120/70 mmHg for nighttime [12].

### 2.4. Transthoracic Echocardiography

TTE recordings were performed with a Vivid E9 GE device with an M5Sc transducer in accordance with the European Association of Cardiovascular Imaging and the American Society of Echocardiography recommendations [13]. Study participants were not merged with other patients; recordings were performed with strict protocol and used solely for presented research. All recordings included at least three cardiac cycles and were digitally stored for analysis by two experienced cardiologists. 2D images were used to assess the following parameters: diastolic thickness of the interventricular septum (IVS), LV end-diastolic dimension (LVEDd), LV end-systolic dimension (LVESd), posterior wall thickness (PW), left atrium diameter (Lad), left atrium volume (LAV), LV mass (LVM), and LV diastolic function parameters. RWT was calculated from the formula: RWT = 2 × PW/LVEDd. LV ejection fraction (LVEF) was assessed using the biplane method of disks (modified Simpson’s rule) based on the formula: LVEF = 100% ∗ ((LV end-diastolic volume − LV end-systolic volume)/LV end-diastolic volume). Linear echocardiographic dimensions were used to calculate the LVM with measurements performed at the end of diastole. A linear 2D derived measurements method was applied. The left ventricle internal dimension, interventricular septum, and posterior wall were measured at end-diastole from 2D recordings in a parasternal long axis view at the level of mitral valve leaflet tips. LVM was calculated from linear dimensions using the formula LVM = 0.6 g + 0.8x{1.04X[(LVIDd + PW + IVSd)^3^ − (LVIDd)^3^]}. LVM was indexed to body surface area (BSA) calculated with the Du Bois formula: BSA = 0.007184 × W^0.425^ × H^0.725^ (W − body weight in kilograms, H − height in meters): LVMI = LVM/BSA. Left atrium volume (LAV) was calculated by the disk summation technique (modified biplane) from the apical four and two chamber views. LAV was indexed to BSA: LAVI = LAV/BSA.

The apical four-chamber view was used to record mitral inflow: early (E) and atrial (A) trans-mitral flow velocities, E/A ratio, and deceleration time of E (DT) were obtained. Isovolumetric relaxation time (IVRT) was acquired by placing the Doppler cursor in the LV outflow tract to display the end of aortic ejection and onset of mitral inflow at the same time. Tissue Doppler imaging in the apical four-chamber view was performed to derive early diastolic mitral annular velocity (e’) from the averaged velocities of the lateral and septal curves. E-to-e’ velocity ratio (E/e’) was calculated. Tricuspid regurgitation velocity (TR) was obtained by color-guided continuous-wave Doppler from the four-chamber apical view. Four diastolic dysfunction criteria were applied, since LVEF was normal in all study participants (>52% for men and >54% for women): (1) LAVI > 34 mL/m^2^, (2) tricuspid regurgitation (TR) velocity > 2.8 m/s, (3) E/e’ > 14, and (4) septal e’ < 7 or lateral e’ < 10 cm/s [14].

### 2.5. Common Carotid Artery Atherosclerotic Plaque and Intima-Media Thickness Assessment

Doppler ultrasound (Vivid E9 GE device with an 11 L transducer) was applied to assess the presence of atherosclerotic plaques (local intima-media thickness of at least 1.2 mm) in the CCA. IMT was measured using echo-tracking technology (GE EchoPAC Clinical Workstation with dedicated software) 1 to 3 cm below the carotid artery bifurcation, where no plaques were present. Measurements were performed on the distal wall of both CCAs along a 1 cm length of a straight segment. IMT was automatically obtained from software at the end of diastole, identified by ECG tracking, and the results were confirmed by an experienced ultrasound operator. The maximum and mean of four IMT measurements were recorded.

### 2.6. Statistical Analyses

Statistical analyses were performed using GraphPad Prism 9.4 software. Selection of statistical tools depended on data distribution, and the Kolmogorov–Smirnov test was used to determine whether it was normal. Accordingly, unpaired Student’s, U Mann–Whitney, and Fisher’s exact tests were used. Correlations were assessed using Pearson and Spearman methods, depending on data distribution; their significance was verified with a dedicated test. Significance was set at <0.05 for all tests. Results are reported as number (percentage, %), arithmetic mean ± standard deviation (SD), and/or median (interquartile range, IQR). Several datapoints were missing and excluded from analyses. Due to marked drug-induced hyperreninemia (more than three times above the upper reference range limit), Ald and DRC were excluded from analysis in six patients, and UAldE in three. Valid ABPM was not available for nine study participants.

## 3. Results

### 3.1. Clinical and Laboratory Data of Study Participants

Among the 63 enrolled participants, there were more women (44) than men (19), the mean age was 61 ± 8.9 years, and BMI 29.1 ± 4. Age, prevalence of T2DM, and the male-to-female ratio were not significantly different between the 26 normotensive and 37 hypertensive patients, while the BMI was higher in the latter (Table 1). Concerning RAAS-interfering medications, five patients with NT used beta-blockers. In hypertensives, the majority was treated with either an ACEI/ARB or a beta-blocker, almost half with a diuretic (but none with a MRA).

Upon testing correlations, in patients with NT, Ald was positively associated with renin: Spearman’s r = 0.4, *p* = 0.047. A stronger correlation in normotensive subjects was recorded after excluding three with T2DM: Pearson’s R = 0.6, *p* = 0.004. 

The same correlation (between Ald and renin) was observed in patients with HT, after excluding those with beta-blocker therapy: Spearman’s r = 0.73, *p* = 0.006 (*n* = 16), as well as after excluding seven subjects with Ald < 3 ng/dL and/or DRC above the upper reference range: r = 0.4, *p* = 0.048 (*n* = 30). These findings suggest that in the recruited population, Ald secretion was renin-dependent to a considerable extent, and not autonomous.

Regarding the laboratory results, there were no statistically significant differences between patients with NT and HT. Only a minority of participants exhibited mild cortisol excess, i.e., in 17%, cortisol was above 50 nmol/L in the 1 mg DST.

With respect to the RAAS, there was a trend toward a higher Ald among patients with HT than NT. We found 16 patients (all female) had an ADRR above 1.2 and an unsuppressed (≥4 ng/dL) Ald, indicative of AAS. Among them, three normotensive (out of seven) and two hypertensive (out of nine) also exhibited MACS. The highest UAldR was 9.7 ug/d; three patients (one with HT) had a value above 8 ug/d; among them, ADRR was positive (1.33) in only one normotensive female (with a DRC = 9.2 mIU/L).

In all subjects, no statistically significant correlations between Ald, DRC, ADRR, UAldE, and demographic parameters (BMI, age), nor other laboratory parameters (UFC, 1 mg DST cortisol, hsCRP, albuminuria, and eGFR) were found. Among women with NT and renin <10 mIU/L, ADRR correlated with BMI: Spearman’s r = 0.61 (*n* = 15).

### 3.2. Ambulatory Blood Pressure Monitoring

Valid ABPM results were available for 54 patients. A majority presented normal BP in the case of patients with NT, and good disease control in those with HT (Table 2).

Surprisingly, negative correlations were found between Ald and: 24 h DBP, daytime SBP and DBP, and nighttime DBP (Spearman’s r between −0.4 and −0.35, *p* between 0.007 and 0.03). Similar results were observed when separately analyzing patients with NT (24 h SBP and DBP, daytime SBP and DBP, nighttime DBP with an r between −0.54 and −0.48), and HT (i.e. 24 h SBP and DBP, daytime SBP and DBP, nighttime DBP, r between −0.54 and −0.42). These correlations may indicate more pronounced Ald release in response to the upright posture (two-hour period preceding baseline morning sample collection as per study protocol) in those with lower BP.

### 3.3. Echocardiographic Parameters and CCA Intima-Media Thickness

The TTE results are presented in Table 3. Similar to ABPM and laboratory data, the parameters of patients with NT did not differ significantly with those of hypertensives.

Based on LVMI, six patients with NT (one male) and seven with HT (all female) had LV hypertrophy (LVMI ≥ 96 and ≥116 g/m^2^ for women and men, respectively), *p* = 0.76.

Since LVEF was normal in all subjects, four criteria were applied in the assessment of diastolic function. Only one patient with NT and two with HT met two of these criteria, while none were found in 11 and 12, respectively. None of the participants met three or four criteria. LAVI was abnormal (≥34 mL/m^2^) in six patients (four with HT). The TR was below 2.8 m/s in all patients.

Upon testing the associations between TTE and the laboratory parameters, LVMI correlated positively with UAldE in non-diabetic subjects: r = 0.34, *p* = 0.01 (*n* = 50), and negatively with DRC in subjects without beta blocker therapy: r = −0.59, *p* = 0.04 (*n* = 38).

No statistically significant associations between the RAAS parameters and IMT, nor differences in hormonal tests between patients with and without an atherosclerotic plaque were found (in the whole sample and in subgroups).

## 4. Discussion

The limitations of the presented study include considerable heterogeneity and the small sample size, which most probably underlie the lack of associations between the hormonal parameters when assessing mineralocorticoid excess and elevated BP, as well as the indicators of pathologic cardiac remodeling, apart from the correlations between LVMI and UAldE, as well as DRC in two subgroups. This is understandable, since an approximate 25% prevalence of AAS (defined as ADRR ≥ 1.2 with concomitant unsuppressed Ald, i.e., >4 ng/dL) in a population of 63 translates to only 16 persons. Furthermore, in subjects with NT and some of those with HT, Ald positively correlated with DRC. 

A further disadvantage from one point of view is that ADRR and 24 h UAldE were determined without modifying chronic RAAS-interfering therapy. On the other hand, it proved to be informative and may be valid in many cases. A shift toward more pragmatic testing for PA should be considered in light of the poor adherence to screening recommendations [7].

Another critical issue is the fact that no PA confirmatory testing was carried out in patients with an elevated ADRR. However, 24 h UAldE and UnaE were acquired in lieu of a formal oral sodium loading test (based on the assumption of high salt intake in the Polish population [15]), which did not reveal a single patient who met a liberal threshold for high UAldE (≥10 ug/d). 

The criteria of AAS proposed here may be considered too permissive by many; yet, adopting an Ald cutoff of 5 ng/dL would not significantly lower the recorded AAS prevalence, since all patients with HT, and five instead of seven with NT, still exhibited a higher Ald. Piaditis et al. reported a 24.1% prevalence of ‘subtle AAS’ among patients with a single adrenal adenoma in a case–control study, in which Ald and DRC cutoffs were established for a DXM-enhanced saline infusion test based on data obtained from controls with NT and normal adrenal glands [16]. A similar (29.4%) biochemically overt PA prevalence was obtained with the same approach by these researchers recently, and, crucially, MRA and adrenalectomy were highly effective in the therapy of HT among PA patients [17]. While our approach of using merely a permissive ADRR to diagnose AAS is perhaps overly simplistic, an assessment of the congruity between these would be valuable (the mean basal ADRR of adrenal adenoma patients was ca. 1.52 ± 0.17 and 1.77 ± 0.39 in the two mentioned studies, respectively). 

In a cross-sectional study by Brown et al., an adjusted prevalence of biochemically overt PA diagnosed by oral salt loading (with a threshold of 10 ug/d) among patients with suppressed PRA was 19.9% in normotensives (*n* = 239), and 27.4% in untreated patients with stage 1 HT (*n* = 89) [2]. The lower UAldE in our subjects may be due to different collection conditions (in-hospital versus ambulatory), populations, medications, etc. A protective effect of ACEIs and ARBs may be in play, even in the case of autonomous Ald excess, since its release is angiotensin II-dependent in many PA patients [8]. Furthermore, as reported above, positive correlations between Ald and renin indicate that most of our patients exhibited no AAS. 

The effect of hypotensive drugs, including beta blockers, might also partly underlie surprising negative correlations between Ald and BP in 24 h monitoring. Their action may have triggered Ald release dependent on ACTH in the morning hours, during which blood was collected.

A correlation between Ald and LVMI implicates the mineralocorticoid in LV hypertrophy in patients without overt PA. More refined measures of abnormal cardiac function (e.g., contractile function assessment by speckle tracking echocardiography) might provide better insight into the adverse effects of AAS.

## 5. Conclusions

While the deleterious effects of overt PA are widely appreciated, AAS is currently not an established disorder; mild PA forms remain largely unrecognized [7]. Our study demonstrates a relatively high prevalence of (possible) autonomous aldosterone excess among patients with adrenocortical adenoma/hyperplasia. The results were obtained without the withdrawal of RAAS-interfering medications. Among others, larger patient samples, assessment before and after hypotensive therapy modification, inclusion of dynamic tests, and longitudinal studies are required to shed more light on the prevalence of AAS and its clinical significance.

Such research has been accumulating in the case of MACS [18]. In our view, in patients with adrenal adenomas, AAS should be considered on par with mild hypercortisolemia, since the clinical consequences of both endocrinopathies may prove equally severe [5].

## Figures and Tables

**Table 1:** Clinical and laboratory data.

	All Patients	Patients with NT	Patients with HT	*p*
*n* (female/male)	63 (44/19)	26 (20/6)	37 (24/13)	0.41
Age [years]	60.5 ± 8.9	60.5 ± 8.8	60.5 ± 9.1	1
BMI [kg/m^2^]	29.1 ± 4	27.9 ± 3.7	30 ± 4.1	0.04
T2DM [*n* (%)]	12 (19%)	3 (11.5%)	9 (24.3%)	0.33
Smokers [*n* (%)]	24 (38.1%)	8 (30.8%)	16 (43.2%)	0.43
Bilateral AI [*n* (%)]	17 (27%)	8 (30.8%)	9 (24.3%)	0.58
Maximal AI dimension [mm]	23.9 ± 11.9	25.7 ± 12.1	22.3 ± 11.5	0.25
Beta blocker therapy [*n* (%)]	25 (39.7%)	5 (19.2%)	20 (54.1%)	0.01
Diuretic therapy [*n* (%)]	11 (17%)	0	12 (32.4%)	<10^−5^
ACEI/ARB therapy [*n* (%)]	22 (35%)	0	23 (62.1%)	<10^−5^
Statin therapy [*n* (%)]	15 (24%)	6 (23.1%)	9 (24.3%)	1
				
eGFR [mL/min/1.73 m^2^]	97 ± 8.4	97.6 ± 7.3	96.5 ± 9.1	0.62
Natrium [mmol/L]	139 (2)	139.5 (3)	139 (2)	0.56
Potassium [mmol/L]	4.3 ± 0.4	4.4 ± 0.4	4.3 ± 0.4	0.52
24 h UNaE [mmol/24 h]	155.9 ± 66	157.2 ± 55.2	154.9 ± 73.6	0.89
1 mg DST cortisol [nmol/L]	23.4 ± 31	0 (38)	0 (41)	0.98
MACS [*n* (%)]	10 (17%)	5 (19.2%)	5 (13.5%)	0.73
24 h UFC [nmol/d]	218.7 ± 140.9	254 ± 151.3	194 ± 129.4	0.1
DHEA-S [ug/dL]	87 ± 64.9	77.1 ± 50.1	93.9 ± 73.4	0.32
Ald [2.52–39.2 ng/dL] *	8.1 ± 4.3	6.2 (3.3)	8.4 (5.5)	0.06
Renin [4.4–46.1 mIU/L] *	14.6 ± 15.8	7.1 (8.4)	12.5 (21.4)	0.08
ADRR [ng/dL/mIU/L] *	1.14 ± 1.17	0.9 (0.8)	0.7 (1.2)	0.3
24 h UAldE [ng/d] **	3.1 ± 2.2	2.1 (2.3)	2.6 (2.3)	0.2
Possible AAS [*n*(%)]	16 (25.4%)	7 (26.9%)	9 (24.3%)	0.78

Legend: * and **—due to hyperreninemia, Ald, DRC and ADRRs of six patients (one normotensive), and UAldE > 8 ng/d of three patients (one with HT) were excluded from analysis; AAS—autonomous aldosterone secretion; ACEI—angiotensin-converting enzyme inhibitor; ADRR—aldosterone-to-direct-renin ratio; AI—adrenal incidentaloma; ARB—angiotensin II receptor blocker; BMI—body mass index; DHEA-S—dehydroepiandrosterone sulfate; DST—overnight dexamethasone suppression test; eGFR—estimated glomerular filtration rate; MACS—mild autonomous cortisol secretion, i.e. cortisol > 50 nmol/L in the overnight 1 mg DST; T2DM—type 2 diabetes mellitus; UAldE—urinary aldosterone excretion; UNaE—urinary natrium excretion.

**Table 2:** Ambulatory blood pressure monitoring results.

	All Patients	Patients with NT	Patients with HT	*p*
*n*	54	22	32	-
24 h SBP [mmHg]	119.7 ± 9.1	117.9 ± 10.4	121.1 ± 8	0.14
24 h DBP [mmHg]	71.6 ± 8	70.4 ± 7.6	72.6 ± 8.4	0.24
Normal dipping [*n* (%)]	40 (72.7%)	17 (77.3%)	23 (71.9%)	0.76
Daytime SBP [mmHg]	123.4 ± 9.3	121.4 ± 10.5	125 ± 8.1	0.1
Daytime DBP [mmHg]	74.8 ± 8.4	73.7 ± 8.2	75.7 ± 8.6	0.31
Nighttime SBP [mmHg]	107.6 ± 9.4	106.2 ± 10.1	108.7 ± 8.8	0.22
Nighttime DBP [mmHg]	61.9 ± 8.4	60.8 ± 7.3	62.8 ± 9.2	0.29

**Table 3:** Echocardiographic and carotid artery intima-media complex data.

	All Patients	Patients with NT	Patients with HT	*p*
*n*	63	26	37	-
IVS [mm]	11 ± 1.5	11 (2.3)	11 (2)	0.45
LVEDd [mm]	45.4 ± 4.2	45 ± 3.7	45.2 ± 4.6	0.82
PW [mm]	10 ± 1.4	10 (2)	10 (2)	0.67
LVEDs [mm]	27.5 ± 2.9	28 (3.5)	28 (4)	0.66
RWT	0.45 ± 0.07	0.45 (0.13)	0.44 (0.13)	0.45
LVM [g]	169 ± 39.3	166.2 ± 42.6	170.9 ± 37.3	0.64
LVMI [g/m2]	88.5 ± 17.9	89 ± 18.9	88.1 ± 17.4	0.86
EF [%]	65.6 ± 6.9	64.8 ± 5.4	67.1 ± 4.6	0.08
LAV [ml]	44.9 ± 17.8	37 (28)	47 (35.5)	0.3
LAVI [ml/m2]	23.5 ± 8.9	20.6 (14.5)	24.6 (18.4)	0.55
E [cm/s]	70 ± 15.5	68.4 ± 15.8	71.1 ± 15.4	0.5
A [cm/s]	81 ± 16.5	79.9 ± 16	81.7 ± 16.9	0.68
E/A	0.89 ± 0.21	0.87 ± 0.18	0.9 ± 0.23	0.64
DT [ms]	192 ± 33.1	193.6 ± 33.7	190.6 ± 33	0.72
Septal e’ [cm/s]	7.84 ± 2.24	8.35 ± 2.53	7.49 ± 2	0.13
Lateral e’ [cm/s]	9.59 ± 2.83	9.73 ± 3.12	9.49 ± 2.64	0.74
Average e’ [cm/s]	8.8 ± 2.11	9 ± 2.4	8.66 ± 1.9	0.53
E/average e′	8.3 ± 2.2	8.2 (2.8)	8.1 (2.3)	0.58
Normal diastolic function [*n* (%)]	60 (95.2%)	25 (96.2%)	35 (94.6%)	1
				
Atherosclerotic plaque [*n* (%)]	19 (42.2%)	6 (22.2%)	13 (35.1%)	0.41
Average IMT [mm]	0.73 ± 0.14	0.7 (0.12)	0.72 (0.15)	0.99
Maximum IMT [mm]	1.01 ± 0.23	0.975 (0.175)	0.98 (0.238)	0.8

## Data Availability

Not applicable.
